# Adapting the WHO package of essential noncommunicable disease interventions, Samoa

**DOI:** 10.2471/BLT.17.203695

**Published:** 2018-06-28

**Authors:** Caroline Bollars, Take Naseri, Robert Thomsen, Cherian Varghese, Kristine Sørensen, Nanne de Vries, Ree Meertens

**Affiliations:** a Department of Health Promotion, Faculty of Health, Medicine and Life Sciences, Maastricht University, PO Box 616, 6200 Maastricht, Netherlands.; b Office of the Director General of Health, Ministry of Health Samoa, Apia, Samoa.; c Department of Management of Noncommunicable Diseases, World Health Organization, Geneva, Switzerland.; d Global Health Literacy Academy, Risskov, Denmark.

## Abstract

**Problem:**

Samoa has been struggling to address the burden of noncommunicable diseases at the health system, community and individual levels.

**Approach:**

The World Health Organization (WHO) package of essential noncommunicable disease interventions for primary health care in low-resource settings was adopted in seven villages throughout Samoa in 2015. The National Steering Committee Members designed and implemented a screening process, and local facilitators and health-care workers collected health and lifestyle data. The WHO/International Society of Hypertension risk assessment was used on villagers older than 40 years to identify people at high risk of noncommunicable disease.

**Local setting:**

Samoa is a small island developing state with increasing morbidity and mortality due to noncommunicable diseases. A national representative survey indicated that 50.1% (595/1188) of the Samoan adult population is at high risk of such diseases. High numbers of noncommunicable diseases are undiagnosed or untreated, because of shortage of health-care staff and lack of awareness of risk factors.

**Relevant changes:**

The teams collected data from 2234 adults. For people older than 40 years, 6.7% (54/804) were identified as being at high-risk and were encouraged to seek treatment or manage risk factors. Community members developed an awareness programme to improve understanding of lifestyle risk factors.

**Lessons learnt:**

Engaging community members was crucial in conducting a successful screening campaign. By identifying those villagers at high risk of developing noncommunicable diseases, early intervention was possible. Education improved awareness of the symptom-free nature of early-stage noncommunicable diseases.

## Résumé

**Problème:**

Les Samoa s'emploient à agir contre la charge que représentent les maladies non transmissibles aux niveaux du système de santé, de la communauté et des individus.

**Approche:**

L'ensemble d'interventions essentielles de l'Organisation mondiale de la Santé contre les maladies non transmissibles pour les soins de santé primaire dans les structures à faibles ressources a été adopté dans sept villages des Samoa en 2015. Les membres du Comité directeur national ont conçu et mis en œuvre un processus de dépistage, et des facilitateurs et prestataires de soins locaux ont collecté des données sur l'état de santé et le mode de vie. L'évaluation des risques de l'OMS/la Société internationale d'hypertension a été utilisée sur les villageois âgés de plus de 40 ans afin d'identifier les personnes exposées à un risque élevé de développer une maladie non transmissible.

**Environnement local:**

Les Samoa sont un petit État insulaire en développement qui affiche des taux croissants de morbidité et de mortalité liés à des maladies non transmissibles. Une enquête représentative à l'échelle nationale a indiqué que 50,1% (595/1188) de la population adulte des Samoa est exposée à un risque élevé de développer une maladie non transmissible. Un grand nombre de maladies non transmissibles ne sont pas diagnostiquées ou ne sont pas traitées en raison d'un manque de personnel de santé et de sensibilisation aux facteurs de risque.

**Changements significatifs:**

Les équipes ont collecté des données auprès de 2234 adultes. Dans les cas des personnes âgées de plus de 40 ans, 6,7% (54/804) ont été identifiées comme étant exposées à un risque élevé et encouragées à se faire soigner ou à maîtriser les facteurs de risque. Les membres de la communauté ont élaboré un programme de sensibilisation afin de mieux faire connaître les facteurs de risque liés au mode de vie.

**Leçons tirées:**

La participation des membres de la communauté s'est avérée essentielle pour mener une campagne de dépistage efficace. L'identification des villageois exposés à un risque élevé de développer une maladie non transmissible a permis une intervention précoce. La sensibilisation a permis de mieux faire connaître la nature asymptomatique des maladies non transmissibles à un stade précoce.

## Resumen

**Problema:**

Samoa se esfuerza por hacer frente a la carga de las enfermedades no contagiosas a nivel del sistema de salud, la comunidad y los individuos.

**Enfoque:**

En 2015 se aprobó en siete aldeas de Samoa el Conjunto de intervenciones esenciales de la Organización Mundial de la Salud (OMS) para las enfermedades no contagiosas para la atención primaria en entornos de bajos recursos. Los miembros del Comité Directivo Nacional diseñaron e implementaron un proceso de evaluación, mientras que los coordinadores locales y los trabajadores de la salud recopilaron datos sobre la salud y el estilo de vida. La evaluación del riesgo de hipertensión de la OMS/Sociedad Internacional de Hipertensión se utilizó en los aldeanos mayores de 40 años para identificar a las personas con un alto riesgo de padecer enfermedades no contagiosas.

**Marco regional:**

Samoa es un pequeño Estado insular en desarrollo con una incidencia cada vez mayor de morbilidad y mortalidad por enfermedades no contagiosas. Una encuesta nacional representativa indicó que el 50,1 % (595/1188) de la población adulta de Samoa tiene un alto riesgo de contraer tales enfermedades. Un elevado número de enfermedades no contagiosas no se diagnostican ni se tratan, debido a la escasez de personal médico y la falta de concienciación sobre los factores de riesgo.

**Cambios importantes:**

El equipo ha recopilado datos de 2234 adultos. Para las personas mayores de 40 años, se identificó que el 6,7 % (54/804) tenían un alto riesgo y se les animó a buscar tratamiento o controlar los factores de riesgo. Los miembros de la comunidad desarrollaron un programa de concienciación para mejorar la comprensión de los factores de riesgo en el estilo de vida.

**Lecciones aprendidas:**

Involucrar a los miembros de la comunidad fue crucial para llevar a cabo una campaña de detección de éxito. Al identificar a los aldeanos que corren un mayor riesgo de contraer enfermedades no contagiosas, es posible la intervención temprana. La educación mejoró la concienciación sobre la ausencia de síntomas de las enfermedades no contagiosas en la fase inicial

## ملخص

**المشكلة:**

ظلت ساموا تكافح للتعامل مع عبء الأمراض غير المعدية على مستوى النظام الصحي والمستوى المجتمعي والمستوى الفردي.

**الأسلوب:**

تم اعتماد مجموعة منظمة الصحة العالمية للتدخلات الأساسية المتعلقة بالأمراض غير المعدية، والخاصة بالرعاية الصحية الأولية في البيئات منخفضة الموارد في سبع قرى في ساموا في عام 2015. وقام أعضاء اللجنة التوجيهية الوطنية بتصميم وتنفيذ عملية فرز، كما قام المسؤولون المحليون وعمال الرعاية الصحية بجمع البيانات الصحية وبيانات نمط الحياة. تم استخدام تقييم المخاطر الخاص بمنظمة الصحة العالمية/الجمعية الدولية لمخاطر ارتفاع ضغط الدم، على القرويين الذين تزيد أعمارهم عن 40 عامًا لتحديد الأشخاص المعرضين لمخاطر عالية من الأمراض غير المعدية.

**المواقع المحلية:**

ساموا هي دولة نامية عبارة عن جزيرة صغيرة مع تزايد حالات الإصابة بالأمراض والوفيات الناجمة عن الأمراض غير المعدية. وأظهر مسح تمثيلي وطني أن 50.1٪ (595/1188) من السكان البالغين في ساموا معرضون لمخاطر عالية من مثل هذه الأمراض. هناك عدد كبير من الأمراض غير المعدية غير المشخصة أو غير المعالجة، بسبب نقص العاملين في مجال الرعاية الصحية وقلة الوعي بعوامل الخطر.

**التغيّرات ذات الصلة:**

قامت الفرق بجمع البيانات من 2234 بالغًا. بالنسبة للأشخاص الذين تزيد أعمارهم عن 40 عامًا، تم تحديد 6.7٪ (54/804) منهم على أنهم معرضون في خطر كبير وتم تشجيعهم على طلب العلاج أو إدارة عوامل الخطر. قام أعضاء المجتمع بتطوير برنامج للتوعية لتحسين فهم عوامل مخاطر نمط الحياة.

**الدروس المستفادة:**

كان إشراك أفراد المجتمع أمرًا حاسمًا في إجراء حملة فرز ناجحة. من خلال التعرف على هؤلاء القرويين المعرضين لخطر كبير للإصابة بالأمراض غير المعدية، كان التدخل المبكر ممكناً. أدى التعليم لتحسين الوعي بطبيعة الأمراض غير المعدية في المراحل المبكرة، والتي تخلو من الأعراض.

## 摘要

**问题:**

萨摩亚一直致力于解决非传染性疾病在社区和个体层面的卫生系统中的负担。

**方法:**

针对资源匮乏地区初级卫生保健的世界卫生组织非传染性疾病基本干预服务包于 2015 年在萨摩亚全国范围内的 7 个村庄中应用。国家指导委员会成员设计并实施了筛查过程，当地协调员和医护人员搜集了卫生和生活方式的相关数据。对 40 岁以上的村民进行世界卫生组织/国际高血压协会风险评估，以确定高风险非传染性疾病患者。

**当地状况:**

萨摩亚是一个小岛国，是发展中国家，其非传染性疾病发病率和死亡率持续增长。一项全国代表性调查表明，萨摩亚 50.1% (595/1188) 的成人患有高风险非传染性疾病。很多非传染性疾病患者由于缺少医护人员且自身缺乏风险因素意识，未能接受诊断或治疗。

**相关变化:**

团队收集了 2234 名成人的数据。40 岁以上的人群中有 6.7% (54/804) 经确认患有高风险疾病，并鼓励他们寻求治疗或管理风险因素。社区成员开发了一项认知计划，以增强人们对生活方式相关风险因素的理解。

**经验教训:**

鼓励社区成员的参与对于成功实施筛查活动来说至关重要。确定患高风险非传染性疾病的村民后，早期干预成为可能。教育增强了人们对非传染性疾病早期无症状性质的认知。****

## Резюме

**Проблема:**

Самоа прилагает все усилия для решения проблемы неинфекционных заболеваний на уровне системы здравоохранения, сообществ и отдельных лиц.

**Подход:**

В 2015 году в семи деревнях на территории Самоа был внедрен пакет основных мероприятий по борьбе с неинфекционными заболеваниями Всемирной организации здравоохранения (ВОЗ) для оказания первичной медико-санитарной помощи в условиях нехватки ресурсов. Члены сообществ разработали и внедрили процесс скринингового обследования, а местные координаторы и работники здравоохранения собрали данные о здоровье и образе жизни. Для выявления среди сельских жителей старше 40 лет людей с высоким риском неинфекционных заболеваний использовалась оценка риска ВОЗ или Международного общества по гипертензии.

**Местные условия:**

Самоа — это небольшое островное развивающееся государство с растущей заболеваемостью и смертностью от неинфекционных заболеваний. Национальное репрезентативное исследование показало, что 50,1% (595/1188) взрослого населения Самоа подвергается высокому риску таких заболеваний. Из-за нехватки медицинского персонала и недостаточной осведомленности о факторах риска при большинстве случаев неинфекционных заболеваний не проводится диагностика или лечение.

**Осуществленные перемены:**

Члены сообществ собрали данные от 2234 взрослых. Среди людей старше 40 лет 6,7% (54/804) были отнесены в группу высокого риска, и им было рекомендовано обратиться за лечением или следить за факторами риска. Члены сообществ разработали программу по повышению осведомленности о факторах риска, связанных с образом жизни.

**Выводы:**

Привлечение членов сообществ имело решающее значение для успешного проведения кампании по использованию скринингового обследования. Выявление среди сельских жителей людей с высоким риском развития неинфекционных заболеваний способствовало более раннему вмешательству. Информирование повысило осведомленность о бессимптомном характере протекания неинфекционных заболеваний на ранней стадии.

## Introduction

More than 90% of premature and largely preventable deaths from noncommunicable diseases, including cardiovascular disease, cancers, chronic lung diseases and diabetes, occur in low- and middle-income countries.^1^ Most premature deaths are linked to the four established risk factors of tobacco use, unhealthy diet, physical inactivity and harmful use of alcohol.^1^ The social burden associated with noncommunicable diseases includes prolonged disability, diminished resources within families and reduced productivity, in addition to tremendous demands on health systems.^2^

The World Health Organization (WHO) designed the package of essential noncommunicable disease interventions for primary health care in low-resource settings as an innovative and action-oriented response to the above challenges.^3^ The four protocols of the package, a prioritized set of cost-effective interventions, which aim to integrate noncommunicable disease care into primary health care,^4^ are: (i) prevention of heart attack, stroke and kidney disease through management of diabetes and hypertension; (ii) health education; (iii) management of asthma and chronic obstructive pulmonary disease; and (iv) assessment and referral of women with breast and cervical cancer.

We describe how the first two protocols of the WHO package were adapted and implemented to suit the local context in Samoa, engaging the community and training local facilitators in the use of early detection and assessment tools, with the aims of: (i) strengthening links between health services and the community; and (ii) meeting the global target of at least 50% of eligible people receiving treatment and counselling through early detection and management of noncommunicable diseases.^5^

## Local setting

Human health resources are constrained in Samoa with a chronic shortage of doctors and nurses; there also exists an inequitable distribution of health professionals and services, with the majority being concentrated in the capital city of Apia. The bulk of patients (70–80%) seen by health professionals have noncommunicable diseases.^6^ A national representative study of the noncommunicable disease risk factors in 2013,^7^ based on the WHO STEPwise approach to surveillance,^8^ indicated that 50.1% (595/1188) of the Samoan adult population is at high risk of developing a noncommunicable disease. The survey found a high incidence of previously undiagnosed and untreated noncommunicable diseases in the country: over 70% of the population had never had their blood pressure (1265/1765) or blood glucose (1315/1765) measured, and only 30–40% of those diagnosed were using appropriate medication.^7^

Identifying those at risk of developing such diseases could prevent the development of serious, debilitating, fatal and/or costly health conditions later in life. Samoa therefore adopted the WHO intervention package at a country level in 2013.^9^ The aims of the *Fa’a Samoa* (Samoan way of life) version of the package were to address key issues in health system delivery, in particular integrating community participation and village outreach services to: (i) ensure early detection of noncommunicable diseases in those at risk; (ii) establish mechanisms for referral to district health facilities for treatment and follow-up; and (iii) increase awareness of risk factors. For the adaptation process, seven villages were selected based on their geographical spread: four from the island of Upolu (Sapulu, Lealalii, Moamoa and Tauo’o) and three from the island of Savaii (Vaisaulu, Lalomalava and Safua). 

## Approach

We adapted the intervention to the Samoan context through the development of a three-step package. The conceptual development of the three-step package took place from September to December 2014, and screening at the pilot sites occurred from February to March 2015. 

Step 1 included consultations with stakeholders to introduce the concept of a *Fa’a Samoa* intervention package. A national steering committee, comprising the health ministry, National Health Service representatives and local WHO Country Office staff, determined the overall direction of the *Fa’a Samoa* package and established national referral criteria for noncommunicable diseases:^10^ symptoms of stroke, hypertension and diabetes; risk factors including smoking, alcohol and inactivity; blood pressure; body mass index; random glucose; cholesterol (total and high-density lipoprotein for those older than 40 years); and WHO/International Society of Hypertension risk prediction.^11^ Specific tools for non-health professionals were developed, including: an assessment community registry form on which to record data; a flowchart for referral of patients demonstrating risks; a follow-up form to record subsequent visits to a health facility; and an awareness project template. All these tools are available from the corresponding author.

Village chiefs nominated one to three representatives of their local Women’s Committee (funded by the Ministry of Women, Community and Social Development) as voluntary local facilitators; their role was to brief other committee members, inform all villagers of planned events and assist with the screening. Village members received information at monthly coordination events and church meetings; the screening itself took place in community halls. Primary health-care staff helped to design the screening algorithm, with a focus on the clinical measurements required at each primary health-care facility.^12^

Step 2 involved training of local facilitators and health-care workers to form cross-disciplinary outreach teams, each comprising four health professionals and six to 10 local facilitators, and the screening process itself. Training sessions took place at local health facilities during January 2015, costing approximately 500 United States dollars per session for refreshments and disposable testing strips for measuring blood sugar and cholesterol. Local facilitators received training in conducting the basic part of the assessment, recording information such as village, name, age, gender, date of birth, contact number, weight (scales), height (stadiometer), known symptoms and details of any risk factors. Health-care workers were educated on the use of WHO/International Society of Hypertension risk prediction charts.

Between February and March 2015, each outreach team screened all village members older than 18 years for indications of elevated risk. Local facilitators recorded the identities of villagers who attended initial screening, inviting those who did not initially attend to a second screening held in May 2015. Health-care workers performed the clinical measurements of blood pressure (an average of two measurements) and random blood glucose and cholesterol levels (using a CardioChek analyser). Health-care workers did not enforce fasting before clinical measurements. For those older than 40 years, the WHO/International Society of Hypertension risk prediction charts were used to estimate the 10-year risk of a major cardiovascular event by using information on age, sex, smoking status, blood pressure, total cholesterol and random blood glucose level (> 11.1 mmol/L indicating diabetes). Risks are provided in the form of scores, with a score of > 30% interpreted as a high risk of noncommunicable disease development.

In Step 3, screening results in terms of numbers (not identities) were delivered to the community at a public meeting and local facilitators were informed of the identities of villagers at high risk. Those villagers were provided with a personalized management plan, including initiation of a treatment regime and/or a risk factor consultation. Members of the Women’s Committees reported to the village chiefs on those villagers and their attendance at follow-up appointments on a quarterly basis, and the steering committee made visits to the community to assess progress.

## Screening results

The teams collected data from 2234 adults between February 2015 and May 2015 (Table 1). Health-care workers measured blood pressure in 1536 adults (70%), of whom 511 (32.7%) had an elevated blood pressure. Of the 1528 adults (68.4%) for whom body mass index (BMI) was measured, 820 (53.7%) had a BMI over 30. For the 1550 villagers (69.4%) for whom random blood glucose data were collected, 108 (7.1%) had elevated levels. Of the screened population older than 40 years, 6.7% (54/806) were classified as being at high risk.

**Table 1:** Health status of the population screened as part of the *Fa’a Samoa* package, Samoa, 2015

Characteristics	No. of people/denominator (%)		
	Male	Female	Total
Total population	2136	1996	4132
Total population > 18 years	1119	1115	2234
**Age, years**			
18–29	339/1119 (30.29)	335/1115 (30.04)	674/2234 (30.17)
30–39	213/1119 (19.03)	234/1115 (20.99)	447/2234 (20.01)
40–49	248/1119 (22.16)	210/1115 (18.83)	458/2234 (20.50)
50–59	157/1119 (14.03)	146/1115 (13.09)	303/2234 (13.56)
60–69	98/1119 (8.76)	121/1115 (10.85)	219/2234 (9.80)
≥ 70	64/1119 (5.72)	69/1115 (6.19)	133/2234 (5.95)
**Symptoms**			
CVD-related symptoms^a^	215/1110 (19.37)	226/1107 (20.42)	441/2217 (19.89)
TIA-related symptoms^b^	231/1111 (20.79)	264/1106 (23.87)	495/2217 (22.33)
Diabetes-related symptoms^c^	199/1105 (18.01)	232/1101 (21.07)	431/2206 (19.54)
**Risk factors**			
Current smoker: smoked tobacco in last 12 months?	500/1109 (45.09)	167/1107 (15.09)	667/2216 (30.1)
Alcohol abuse: binge drinking, excessive weekly intake	456/1105 (41.27)	74/1102 (6.72)	530/2207 (24.01)
Physical inactivity: less than 30 minutes of exercise 3 times a week	606/1097 (55.24)	614/1101 (55.77)	1220/2198 (55.51)
**Health status**			
Systolic blood pressure ≥ 140 mmHg	277/732 (37.84)	234/831 (28.16)	511/1563 (32.69)
BMI, kg/m^2^			
< 18.5	41/719 (5.70)	61/809 (7.54)	102/1528 (6.68)
18.5–24.9	148/719 (20.58)	90/809 (11.12)	238/1528 (15.58)
25–29.9	211/719 (29.35)	157/809 (19.41)	368/1528 (24.08)
30–34.9	170/719 (23.64)	187/809 (23.11)	357/1528 (23.36)
≥ 35	149/719 (20.72)	314/809 (38.81)	463/1528 (30.30)
Random blood glucose ≥ 11.1mmol/L	36/723 (4.98)	72/827 (8.71)	108/1550 (6.97)
Total cholesterol, mmol/L			
< 6.2	256/290 (88.28)	274/378 (72.49)	530/668 (79.34)
6.2–7.99	22/290 (7.59)	79/378 (20.90)	101/668 (15.12)
≥ 8	12/290 (4.14)	25/378 (6.61)	37/668 (5.54)
HDL cholesterol < 1 mmol/L (male), < 1.3 mmol/L (female)	169/281 (60.14)	231/369 (62.6)	400/650 (61.54)
WHO/ISH CVD risk assessment			
< 10%	285/374 (76.2)	353/432 (81.71)	638/806 (79.16)
10–19.9%	46/374 (12.3)	40/432 (9.26)	86/806 (10.67)
20–29.9%	15/374 (4.01)	13/432 (3.01)	28/806 (3.47)
30–39.9%	12/374 (3.21)	7/432 (1.62)	19/806 (2.36)
≥ 40%	16/374 (4.28)	19/432 (4.4)	35/806 (4.34)

BMI: body mass index; CVD: cardiovascular disease; HDL: high-density lipoproteins; ISH: International Society of Hypertension; TIA: transient ischaemic attack; WHO: World Health Organization.

^a^ Symptoms are chest pain, tightness and/or breathlessness, likely to be worsened by exercise.

^b^ Symptoms, which may be permanent or transient, are left- or right-sided weakness of limbs or face, difficulty speaking or periods of resolving blindness.

^c^ Symptoms include constant thirst/drinking/passing urine, frequent bacterial infection (urinary tract, chest and skin), tiredness, blurred vision and foot ulcers.

Notes: Differences between total numbers of participants and numbers for which clinical measurements are available are due to missing data and the limited numbers of workers at the primary health-care level.

Since the 2011 Population and Housing Census Report from Samoa^13^ only provides stratified age group data by statistical region and not by village, we extrapolated the population data to calculate the proportion of the population per village aged over 18 years. Differences in the total numbers of participants are due to missing clinical assessment data, a result of the limited number of health-care workers at the primary level.

## Relevant changes

As a result of the *Fa’a Samoa* intervention package, those high-risk members of the population were made aware of their risk status and were motivated to seek treatment or manage risk factors. Village members learned about the risk of noncommunicable diseases at organized sessions, which brought villagers together and translated findings into their language and understanding. By being made aware that such diseases have no symptoms in their early stages, the cultural belief that illness is only present when a person feels ill was overcome.

After initiation by the steering committee and discussions with the local facilitators, village members chose to develop a noncommunicable disease awareness project. Data from the screening focused the village members on prevention of noncommunicable diseases by, for example, reducing intake of salt, sugar, tobacco and/or alcohol.

## Lessons learnt

The *Fa’a Samoa* intervention package used a community-focused, participatory approach and strengthened links between health services and those they serve: their communities (Box 1). Successful application of the package was demonstrated in less than a year and results are consistent with the national survey conducted in 2013.^7^ The health screening conducted in the villages enabled the detection of people at high risk of developing noncommunicable diseases and raised awareness of disease risk factors through the local facilitators.

Box 1 **Summary of main lessons learnt**

- The *Fa’a Samoa* package strengthened links between health services and their communities through application of a community-focused, participatory approach.
- Data management and follow-up of people referred to the health-care facility could be improved to monitor progress of the intervention over time.
- Providing the screening results to the community improved understanding of the risks of such diseases, increasing awareness of the symptom-free nature of their early stages.

Critical to this result was the clearly defined three-step *Fa’a Samoa* package process, including thresholds for assessing risk as defined by national referral criteria, and training both health-care workers and local facilitators in the use of data recording and patient monitoring tools. To scale up to a nation level, including the community, would ensure compliance and help to address the challenges within the health-care system. Although the lack of a digitalized health system in Samoa hindered the collection of data, improvements in data management and the follow-up of those referred could improve adherence to recommended treatment.

Design of the *Fa’a Samoa* package was based upon existing systems without the need to mobilize additional resources. Replicating the *Fa’a Samoa* package in other Samoan villages should be feasible without any drain on resources. Village members at high risk of developing noncommunicable diseases can then be identified by their local health facility and receive effective interventions to prevent their condition from progressing to a more serious illness.^14^

The *Fa’a Samoa* package provides an example of the transformation of the health system structure by incorporating communities. We have also highlighted the relevance of training primary health-care workers and using local community stakeholders in the early detection of noncommunicable diseases. Future community-based interventions could accelerate progress towards the goal of universal health coverage, as detailed in the Tokyo Declaration on Universal Health Coverage.^15^

## References

[1] Global action plan for the prevention and control of NCDs 2013-2020. Geneva: World Health Organization; 2013. Available from: http://www.who.int/nmh/events/ncd_action_plan/en/ [cited 29 May 2018].

[2] World Economic Forum and Harvard School of Public Health. The Global Economic Burden of NCDs. Geneva: World Health Organization; 2011. Available from: http://apps.who.int/medicinedocs/documents/s18806en/s18806en.pdf [cited 29 May 2018].

[3] Package of essential noncommunicable disease interventions for primary health care in low-resource settings. Geneva: World Health Organization; 2010.

[4] Mendis S, Chestnov O. Policy reform to realize the commitments of the political declaration on noncommunicable diseases. Br Med Bull. 2013;105(1):7–27. 10.1093/bmb/ldt001 23571458

[5] NCD Global Monitoring Framework. Geneva: World Health Organization; 2013. Available from: http://www.who.int/nmh/global_monitoring_framework/en/ [cited 2018 Jun 11].

[6] Assessment of primary health care situation in Samoa prior to PEN implementation. Apia: Samoan Ministry of Health; 2014.

[7] Samoa STEPS. NCD risk factor STEPS Report. Apia: Samoan Ministry of Health; 2014 Available from: http://www.health.gov.ws/images/uploadPDFs/HealthSurveysStat/SamoaSTEPSReport2014.pdf [cited 12 June 2018].

[8] STEPwise approach to surveillance: a standardized method for collecting, analysing and disseminating data in WHO member countries. Geneva: World Health Organization; 2002. Available from: http://www.who.int/ncds/surveillance/steps/en/ [cited 29 May 2018].

[9] Implementation tools: Package of essential noncommunicable (PEN) disease interventions for primary health care in low-resource settings. Geneva: World Health Organization; 2013. Available from: http://apps.who.int/iris/bitstream/handle/10665/133525/9789241506557_eng.pdf?sequence=1 [cited 29 May 2018].

[10] Samoa Bureau of Statistics. Samoa socioeconomic atlas. Apia: Government of Samoa; 2011.

[11] WHO/ISH cardiovascular risk prediction charts. Geneva: World Health Organization; 2007. Available from: http://www.who.int/cardiovascular_diseases/guidelines/Questions%20and%20Answers%20on%20web%20PDF%20file%2012.9.2007.pdf?ua=1 [cited 2018 Jun 11].

[12] Tools for implementing WHO PEN. Planning, implementation and assessment. Geneva: World Health Organization; 2014. Available from: http://www.who.int/ncds/management/pen_tools/en/ [cited 29 May 2018].

[13] Samoa Bureau of Statistics. Population and housing census 2011. Apia: Government of Samoa; 2011.

[14] Rothenberg RB, Potterat JJ, Woodhouse DE. Personal risk taking and the spread of disease: beyond core groups. J Infect Dis. 1996 10;174 Suppl 2:S144–9. 10.1093/infdis/174.Supplement_2.S144 8843244

[15] Tokyo Declaration on Universal Health Coverage: all together to accelerate progress towards UHC. Geneva: World Health Organization; 2017. Available from: http://www.who.int/universal_health_coverage/tokyo-decleration-uhc.pdf [cited 2018 Jun 11].

